# Concern about the risk of aerosol contamination from ultrasonic scaler: a systematic review and meta-analysis

**DOI:** 10.1186/s12903-024-03996-2

**Published:** 2024-04-05

**Authors:** Priscilla Gonçalves Lomardo, Mariana Campello Nunes, Patrícia Arriaga, Lívia Azeredo Antunes, Aldir Machado, Valquiria Quinelato, Telma Regina da Silva Aguiar, Priscila Ladeira Casado

**Affiliations:** 1 Department of Periodontology, School of Dentistry, Fluminense Federal University, Rua Mário Santos Braga, nº 28, Centro, Niterói, Rio de Janeiro CEP 24040-110 Brazil; 2 Department of Specific Information, School of Dentistry, Fluminense Federal University, Dr. Silvio Henrique Braune Street, 22 – Centro, Nova Friburgo, RJ 28625- 650 Brazil; 3 National Institute of Traumatology and Orthopedics, Research department, Rio de Janeiro, Brazil

**Keywords:** COVID-19, Ultrasonic scaler, Aerosols, Environmental contamination, Bioaerosol, SARS-CoV-2 virus

## Abstract

**Background:**

Many instruments used in dentistry are rotary, such as handpieces, water syringes, and ultrasonic scalers that produce aerosols. The spray created by these instruments can carry, in addition to water, droplets of saliva, blood, and microorganisms, which can pose a risk of infections for healthcare professionals and patients. Due to the COVID-19 pandemic, this gained attention.

**Objective:**

The aim was to carry out a systematic review of the evidence of the scope of the aerosol produced by ultrasonic scaler in environmental contamination and the influence of the use of intraoral suction reduction devices.

**Design:**

Scientific literature was searched until June 19, 2021 in 6 databases: Pubmed, EMBASE, Web of science, Scopus, Virtual Health Library and Cochrane Library, without restrictions on language or publication date. Studies that evaluated the range of the aerosol produced by ultrasonic scaler during scaling/prophylaxis and the control of environmental contamination generated by it with the use of low (LVE) and high (HVE) volume evacuation systems were included.

**Results:**

Of the 1893 potentially relevant articles, 5 of which were randomized controlled trials (RCTs). The meta-analysis of 3 RCTs showed that, even at different distances from the patient’s oral cavity, there was a significant increase in airborne bacteria in the dental environment with the use of ultrasonic scaler. In contrast, when meta-analysis compared the use of HVE with LVE, there was no significant difference (*P* = 0.40/CI -0.71[-2.37, 0.95]) for aerosol produced in the environment.

**Conclusions:**

There is an increase in the concentration of bioaerosol in the dental environment during the use of ultrasonic scaler in scaling/prophylaxis, reaching up to 2 m away from the patient’s mouth and the use of LVE, HVE or a combination of different devices, can be effective in reducing air contamination in the dental environment, with no important difference between different types of suction devices.

## Background

Every aspect of life has been influenced by an outbreak of the new coronavirus disease (COVID-19) in China [1] which greatly changed the routine in dental clinics. Due to the COVID-19 pandemic, the Centers for Disease Control and Prevention (CDC) recommended in principle, avoiding aerosol-generating procedures in the dental environment whenever possible, not using equipment that produces aerosols, and prioritizing the use of hand instruments only [2].

However, many of the instruments used in dentistry are rotary, such as handpieces, water syringes, and ultrasonic scalers. The spray created by these instruments can carry, in addition to water, droplets of saliva, blood, and microorganisms, which can pose a risk of infections for healthcare professionals and patients [3].

Particles formed by liquids and solids dispersed and suspended in the air are aerosols, which become bioaerosols when microorganisms excreted by the body dissolve with the aerosols through the act of coughing, breathing vigorously, sneezing, or speaking loudly [4, 5].

Micik et al. used the terms “aerosol” and “splatter” in 1969, in which they were defined as particles smaller and larger than 50 micrometers (µm) in diameter, respectively. The first term refers to small particles that remain in the air for a period of time before being deposited on surfaces or entering the airways. The second are particles or droplets that are forcibly ejected from the operating site, reaching a trajectory similar to that of a bullet until they come into contact with a surface or fall to the ground [6].

With a simulation using computational fluid dynamics to quantify the transport of large droplets and aerosols in dental clinic environments, we better understand the risks associated with a common dental procedure such as ultrasonic scaling. Aerosols below 15 μm remain in the air for up to 7.13 min on average and can travel up to 25.45 m on average from their source, potentially contaminating entire clinics [3].

The water spray droplets produced during ultrasonic scaling are extremely light in weight and release large numbers of microorganisms into the air [7]. The bacterial challenge appears to be considerable and it is likely that viruses and bacteria can be spread in this way [8]. It should also be taken into account that particles ranging from 0.3 to 5 *µm* increase significantly after instrumentation with an ultrasonic scaler [9] and the variation in ultrasonic frequency causes an increase in surface contamination, as well as the type of suction used, influences the degree of contamination [10].

Due to the fact that ultrasonic scaler is one of the equipment that produces the most aerosol and can be responsible for spreading the SARS-CoV-2 virus during dental care, which is a major concern among dentists, especially periodontists, this work aimed to carry out a systematic review of the evidence of the reach of the aerosol, produced by ultrasonic scaler during scaling and prophylaxis, in the contamination of the dental environment and the influence of the use of intraoral suction devices in the reduction of this contamination.

## Materials and methods

The present systematic review was registered in the PROSPERO (International Prospective Register of Systematic Reviews) [11] under the number #CRD42020191209 and conducted in accordance with the recommendations of the “Cochrane Handbook for Systematic Reviews of Interventions” [12] and following the guidelines of the PRISMA checklist [13]. Clinical questions were organized using the “PECO” (Population, Exposition, Comparison and Outcome) strategy.

### Objective

The objective of this study was to carry out a systematic review of the evidence of the reach of the aerosol in distance traveled, produced by ultrasonic scaler during scaling and prophylaxis, in the contamination of the dental environment and the influence of the use of intraoral suction devices in the reduction of this contamination.

### Focus question

What is the evidence of the reach of the aerosol in distance traveled produced by scaling with ultrasonic scaler in the contamination of the dental environment and the influence of intraoral suction devices in the reduction of this contamination?

### Search strategy

Scientific literature was searched in six electronic databases until June 19, 2021 through Pubmed (https://pubmed.ncbi.nlm.nih.gov), EMBASE (https://www.embase.com), Web of Science (www.webofscience.com), Scopus (www.scopus.com), Virtual Health Library (VHL - in the LILACS, BBO, and IBECS databases) (bvsalud.org), and Cochrane Library (www.cochranelibrary.com). No restrictions on language or publication date were imposed. In addition to the electronic search, a manual search was performed using the reference lists of the selected articles. In addition, information was searched in the OpenGrey open access database [14] for unpublished studies (grey literature) using the same terms.

The following MeSH terms (Medical Subjects Headings) [15] were used for the search: “dental care”, “dental prophylaxis”, “ultrasonic therapy”, “dental scaling” and “aerosols”. In addition, other synonyms of DeCS (Health Sciences Descriptors) [16] and free terms were applied in the search, they are: “delivery of dental care”, “dental treatment”, “ultrasonic instrumentation”, “ultrasonic dental scale”, “ultrasonic scaling”, “dental cleaning”, “subgingival scaling”, “supragingival scaling”, “splatter”, “aerosol contamination”, “bioaerosol”, “bio-aerosol”, “airborne”, “dental aerosols”. All descriptors were connected through the Boolean operators “AND” and “OR”. The search strategy is described in Tables 1 and 2. The Endnote web software was used to organize the studies [17].

**Table 1:** Search strategy associated to Population and Exposition

Search strategy									
Population		#1	*(dental care [MeSH] OR dental prophylaxis [MeSH] OR ultrasonic therapy [MeSH] OR dental scaling [MeSH] OR delivery of dental care OR dental treatment OR Ultrasonic instrumentation OR ultrasonic dental scale OR ultrasonic scaling OR dental cleaning OR subgingival scaling OR supragingival scaling)*						
Exposition		#2	*(aerosols [MeSH] OR Splatter OR aerosol contamination OR bioaerosol OR bio-aerosol OR airborne OR dental aerosols)*						
Search combination		#1 AND #2							

**Table 2:** Specific search strategy for each Database

Database	Search strategy
**Pubmed**	**#1** “dental care“[MeSH Terms] OR “dental care“[Title/Abstract] OR “delivery of dental care“[Title/Abstract] OR “dental treatment“[Title/Abstract] OR “ultrasonic therapy“[MeSH Terms] OR “ultrasonic therapy“[Title/Abstract] OR “ultrasonic scale*“[Title/Abstract] OR “ultrasonic instrumentation“[Title/Abstract] OR “ultrasonic dental scale*“[Title/Abstract] OR “ultrasonic scaling“[Title/Abstract] OR “dental prophylaxis“[MeSH Terms] OR “dental prophylaxis“[Title/Abstract] OR “dental cleaning“[Title/Abstract] OR “dental scaling“[MeSH Terms] OR “dental scaling“[Title/Abstract] OR “subgingival scaling“[Title/Abstract] OR “supragingival scaling“[Title/Abstract]
	**#2** “aerosols“[MeSH Terms] OR “aerosols“[Title/Abstract] OR “splatter“[Title/Abstract] OR “aerosol contamination“[Title/Abstract] OR “bioaerosol“[Title/Abstract] OR “bio-aerosol“[Title/Abstract] OR “airborne“[Title/Abstract] OR “dental aerosols“[Title/Abstract]
	#1 AND #2
**Web of Science**	#1 “dental care” OR “Delivery of dental care” OR “dental treatment” OR “ultrasonic therapy” OR “ultrasonic scale*” OR “ultrasonic instrumentation” OR “ultrasonic dental scale*” OR “ultrasonic scaling” OR “dental prophylaxis” OR “dental cleaning” OR “dental scaling” OR “subgingival scaling” OR “supragingival scaling”
	#2 aerosols OR splatter OR “aerosol contamination” OR bioaerosol OR bio-aerosol OR airborne OR “dental aerosols”
	#1 AND #2
**Scopus**	#1 TITLE-ABS-KEY ( ( “dental care” OR “Delivery of dental care" OR “dental treatment" OR “ultrasonic therapy" OR “ultrasonic scale*" OR “ultrasonic instrumentation" OR “ultrasonic dental scale*" OR “ultrasonic scaling" OR “dental prophylaxis" OR “dental cleaning" OR “dental scaling" OR “subgingival scaling" OR “supragingival scaling" ) )
	#2 TITLE-ABS-KEY ( ( aerosols OR splatter OR “aerosol contamination" OR bioaerosol OR bio-aerosol OR airborne OR “dental aerosols" ) )
	#1 AND #2
**BVS**	#1 mh:“dental care” OR “dental care” OR “Delivery of dental care” OR “dental treatment” OR mh: “ultrasonic therapy” OR “ultrasonic therapy” OR “ultrasonic scale*” OR “ultrasonic instrumentation” OR “Ultrasonic dental scale*” OR “ultrasonic scaling” OR mh:“dental prophylaxis” OR “dental prophylaxis” OR “dental cleaning” OR mh:“dental scaling” OR “dental scaling” OR “subgingival scaling” OR “supragingival scaling”
	#2 mh:aerosols OR aerosols OR splatter OR “aerosol contamination” OR bioaerosol OR bio-aerosol OR airborne OR “dental aerosols”
	#1 AND #2
**Embase**	#1 dental procedure’/exp/mj OR ‘dental procedure’:ab,ti OR ‘delivery of dental care’:ab,ti OR ‘ultrasound therapy’/exp/mj OR ‘ultrasound therapy’:ab,ti OR ‘ultrasonic scaler’/exp/mj OR ‘ultrasonic scaler’:ab,ti OR ‘ultrasonic instrumentation’:ab,ti OR ‘ultrasonic dental scale*’:ab,ti OR ‘dental prophylaxis’/exp/mj OR ‘dental prophylaxis’:ab,ti OR ‘dental scaling’/exp/mj OR ‘dental scaling’:ab,ti OR ‘subgingival scaling’:ab,ti OR ‘supragingival scaling’:ab,ti
	#2 ‘aerosol’/exp/mj OR aerosol:ab,ti OR splatter:ab,ti OR ‘aerosol contamination’:ab,ti OR ‘bioaerosol’/exp/mj OR bioaerosol:ab,ti OR ‘bio aerosol’:ab,ti OR ‘airborne particle’:ab,ti
	#1 AND #2
**Cochrane**	#1 MeSH descriptor: [Dental Care] explode all trees OR delivery of dental care OR dental treatment OR MeSH descriptor: [Ultrasonic Therapy] explode all trees OR ultrasonic scale* OR ultrasonic instrumentation OR ultrasonic dental scale* OR ultrasonic scaling OR MeSH descriptor: [Dental Prophylaxis] explode all trees OR dental cleaning OR MeSH descriptor: [Dental Scaling] explode all trees OR subgingival scaling OR supragingival scaling
	#2 MeSH descriptor: [Aerosols] explode all trees OR splatter OR aerosol contamination OR bioaerosol OR bio-aerosol OR airborne OR dental aerosols
	#1 AND #2

### Selection criteria

The eligibility requirements were outlined according to the PECOS strategy:

P (Population of interest): patients undergoing dental scaling treatment with ultrasonic scaler were included;

E (Exposure): Aerosol produced by ultrasonic scaler;

C (Comparison): comparison of contamination reduction with the use of different intraoral suction devices;

O (Outcome): contamination of the environment caused by aerosol from ultrasonic scaler;

S (Study design): randomized controlled trials (RCT) were included.

Exclusion criteria were studies that use manual scaling; studies that included prior use of mouthwashes and studies that used external air decontamination systems.

The eligibility requirements considered for studies to be included in this review were: human studies; studies that evaluated the range of the aerosol produced by ultrasonic during scaling procedures; studies that evaluated the contamination of the environment by the aerosol produced by dental ultrasonic and studies that used intraoral suction reduction devices to control the aerosol.

### Screening process

At first, two reviewers (PGL and MCN) independently selected titles and abstracts. Disagreements were resolved through discussion with a third reviewer (TRSA). Studies that appeared to meet the inclusion criteria or that did not have sufficient information in their titles and abstracts were selected for evaluation of the full article at a later stage. The same reviewers independently assessed full texts to determine whether studies were eligible. Data extraction and risk of bias were performed in studies that met the inclusion criteria.

### Data extraction

All data were extracted individually by two reviewers (PGL and MCN) and discrepancies were discussed by a third reviewer (TRSA). Reviewers were calibrated in applying the inclusion and exclusion criteria applied to a sample of 20% of the studies to determine inter-rater agreement (Kappa = 0.80). All necessary data were found in the studies, and it is not necessary to contact the authors for clarification.

The synthesis of the extracted data was organized in table with the following variables: first author, year of publication, country of origin, type of clinic, patient involved, type of suction device, collected distances, type of incubation, outcome measure and results.

### Outcome measures

The outcome measure was the count of bacterial colony forming units (CFU) present in the oral aerosol, produced by ultrasonic scaler during scaling and prophylaxis, collected through plates with culture media positioned at different distances around the patient and/or the clinic.

### Assessment of the risk of bias and quality

The quality assessment of the studies was performed by the same reviewers (PGL and MCN) independently and any disagreement between them was resolved through consultation with a third party (TRSA).

The Cochrane Collaboration Tool was used to assess the risk of bias using the updated Risk of Bias 2 (RoB 2) tool [18].

This tool evaluates five domains that can be classified as: low risk of bias, some concerns or high risk of bias. The domains are:

D1: Randomization process;

D2: Deviations from intended interventions;

D3: Missing result data;

D4: Measurement of the result; and.

D5: Selection of reported result.

This tool also allows for ranking the overall risk of bias, which receives the least favorable ranking among the assessed risks for the domains. The judgment about the risk of bias resulting from each domain is proposed by an algorithm, based on signaling questions, which help the reviewer to assess the important factors for the evaluation of each domain.

### Statistical analysis

Meta-analyses were performed using the Review Manager software, Version 5.4.1 (Nordic Cochrane Center, Cochrane Collaboration) [19]. A meta-analysis of the reach of the aerosol in the contamination of the environment and a meta-analysis of the reduction of the contamination of the environment were performed, comparing the use of high-volume evacuation (HVE) and low-volume evacuation (LVE), both expressed in mean and standard deviation of CFU/m³. The inverse variance statistical method was used, with a random effects analysis model. Forest plots were calculated for 95% confidence intervals (CI) and *P* values. Heterogeneity between study results and quantification of inconsistency was assessed using the I^2^ test. Results were expressed as standardized mean difference. Subgroups were established according to the distance of the aerosol reach in relation to the patient’s oral cavity.

### Analysis of certainty of evidence

The quality of evidence (certainty in effect estimates) was analyzed by two reviewers (PGL and PA) using the assessment, development and assessment of recommendations (GRADE) approach [20]. The domains evaluated in clinical studies were: risk of bias, inconsistency, indirectness, imprecision and publication bias.

GRADE defines the quality of scientific evidence in a clearer and more objective way, and can be classified as high, moderate, low or very low.

## Results

### Selection of studies

**Fig. 1:**
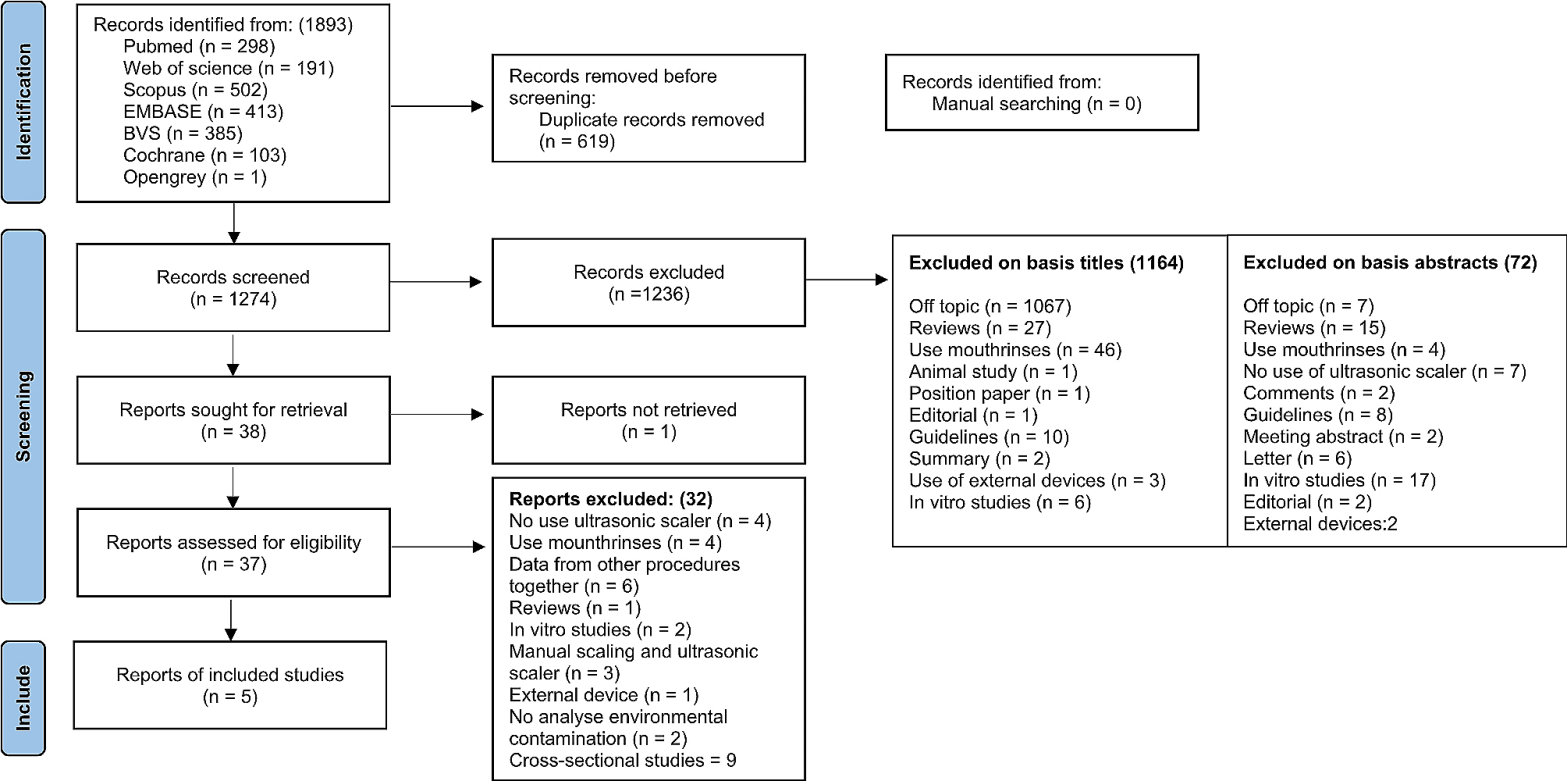
PRISMA 2020 flow diagram (13) of the screening and selection process

A total of 1893 relevant records were found: 298 references from Pubmed, 191 from Web of Science, 502 from Scopus, 413 from EMBASE, 385 from VHL, 103 from Cochrane Library and 1 from Opengrey. 619 duplicate references were removed; 1274 studies were analyzed by title and abstract; 1236 were excluded after this selection; and 38 studies were selected for full-text analysis. Among the 38 selected studies, 33 studies were excluded. Figure 1 outlines the search process and reasons for exclusions. Five randomized controlled trials (RCTs) were included. The synthesis of the extracted data was organized in the Table 3.

**Table 3:** Main characteristics of the included studies

	Country	Type of clinic	Patient involved	Type of sucton device	Collected distances	Type of incubation	Outcome measure		Result			
								Baseline	While using ultrasonic scaler			
**Randomized controlled trials**												
**Desarda, 2014**	India	Single chair	Periodontal disease	High-volume evacuation	12 e 20 inches (30 e 50 cm)	Aerobics	CFU	Uninformed	LVE	11,08 ± 2,25		
									HVE	12,14 ± 1,93		
**Holloman, 2015**	USA	Single chair	Healthy	*Isolite* with high-volume and low-volume evacuations	6 inches (15,24 cm)	Anaerobic	CFU/mL	0		During exposure	After exposure	
									LVE	3,30 ± 0,88	1,65 ± 1,15	
									Combination system	3,61 ± 0,95	2,00 ± 1,17	
**King, 1997**	USA	Single chair	Healthy	Modified high-volume evacuation	6 inches (15,24 cm)	Aerobics	CFU					
								0,16 ± 0,23	LVE	45,13 ± 28,95		
									HVE	2,63 ± 3,62		
**Suprono, 2021**	USA	Multichair	Healthy	High-volume evacuation	<2,0 feet (60 cm); 2–4 feet e > 4 feet (120 cm)	Aerobics	CFU			Zone 1	Zone 2	Zone 3
								1,84 ± 1,68	HVE	441,61 ± 569,17	4,88 ± 2,96	5,47 ± 3,58
									Combination system	158,71 ± 331,55	1,94 ± 1,61	3,00 ± 1,77
**Timmerman, 2004**	Netherlands	Single chair	Periodontal disease	High-volume and low –volume evacuations	40 e 150 cm	Aerobics and anaerobic	CFU			40 cm	150 cm	
								0,2 ± 0,4	LVE	4,3 ± 3,5	10,3 ± 9,5	
									HVE	2,0 ± 1,4	8,1 ± 11,3	

### Study characteristics

The studies originated from 3 different countries: 1 from India [21], 3 from the United States [22–24] and 1 from Netherlands [25]. All selected articles were written in English. All included studies used ultrasonic scaler during treatments, but not all specified the type used, whether piezoelectric, magnetostrictive or sonic. In addition, they used high-volume and/or low-volume suction devices and made comparisons between two types of suction. Four studies were carried out in a dental environment with a single and only chair [21–23, 25], and one study used a multi-chair environment [24].

Two studies had as sample patients diagnosed with periodontal disease [21, 25]. The other studies were considered as if they had evaluated periodontally healthy patients [22–24].

All the studies measured aerosol contamination using colony forming units (CFU). The culture medium varied between studies. Some studies used culture media for aerobic and anaerobic bacteria [25], others only for aerobic bacteria culture [21, 23, 24], and one study only used anaerobic culture [22].

### Assessment of risk of bias and quality

**Fig. 2:**
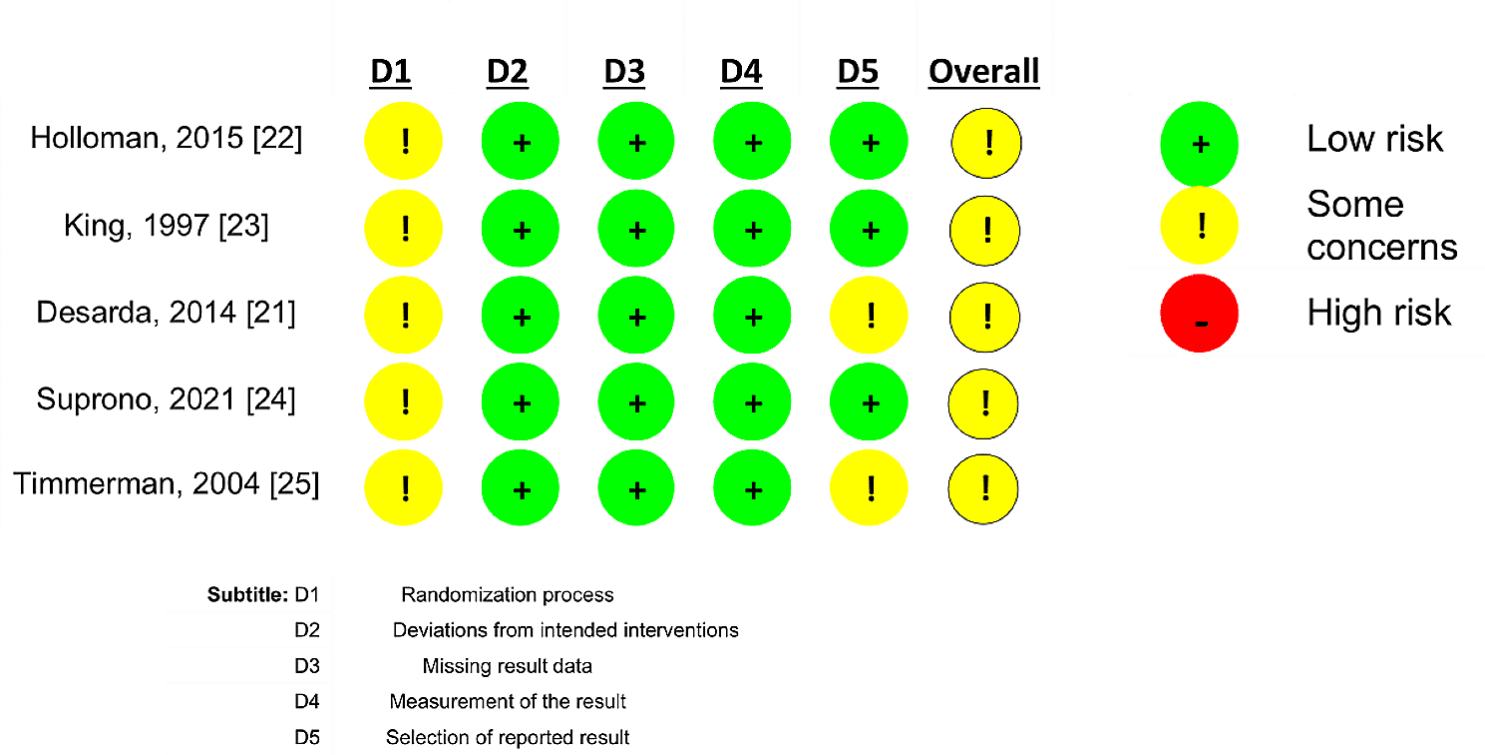
Risk of bias in randomized controlled trials analyzed using the RoB 2 tool

The quality of randomized controlled trials is shown in Fig. 2. None of the randomized controlled trials scored the highest in the quality analysis. The five studies did not describe the allocation sequence and two [21, 25] did not analyze the data according to a pre-specified analysis plan. Thus, they were characterized as some concerns.

### Meta-analysis

**Fig. 3:**
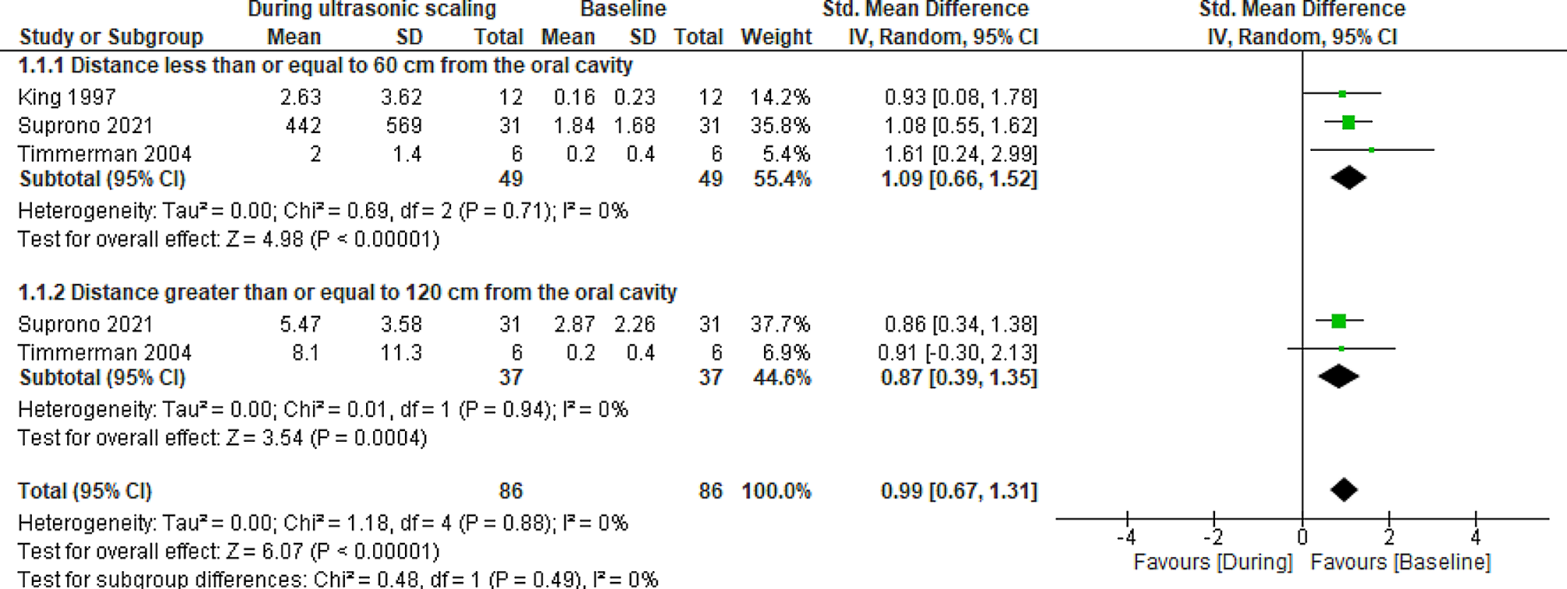
Analysis 1 – Contamination of the dental environment by aerosol produced during scaling with ultrasonic, in randomized controlled studies. Primary outcome: contamination of the dental environment. Subtitle: SD: Standard deviation, CI: Confidence interval

**Fig. 4:**
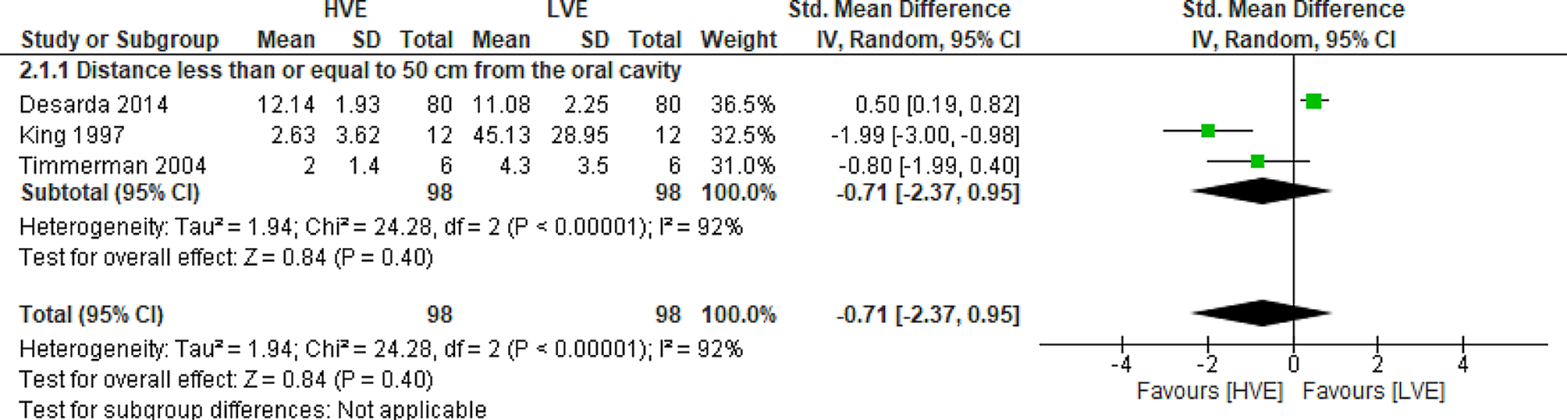
Analysis 2 - Reduction in the level of aerosol contamination by comparing the use of high (HVE) and low (LVE) volume suction in randomized controlled trials. Secondary outcome: reduction in the level of contamination. Subtitle: SD: Standard deviation, CI: Confidence interval

Two meta-analyses of RCTs were performed. The first in relation to contamination of the environment before and during the use of ultrasonic scaler and the second, referring to the reduction of contamination when comparing the use of high-volume suction versus low-volume suction. The analyzes carried out, taking into account the primary outcome of contamination of the dental environment, are shown in Fig. 3, and the secondary outcome related to the reduction of contamination of the environment, comparing the use of HVE and LVE, is shown in Fig. 4.

Only three randomized controlled studies [23–25] were included in the meta-analysis of data on environmental contamination and were divided into two subgroups: one considering data at a distance of less than or equal to 60 cm and another at a distance greater than or equal to 120 cm. For the contamination reduction analysis comparing different types of suction, data from three RCTs [21, 23, 25] were also included.

In the meta-analysis of the RCTs [23–25] (Fig. 3), the studies were homogeneous and indicated that, both at a distance less than or equal to 60 cm and at a distance greater than or equal to 120 cm from the patient’s oral cavity, there is disclosure of a smaller amount of bacteria in the dental environment before the ultrasonic procedure, even though high-volume suction was used, showing a significant increase in bacteria in the air in the dental environment with the use of ultrasonic scaler (*P* < 0.00001/CI 0.99, [0.67, 1.31]), quantifying the magnitude of the effect, according to the Cohen scale [26], in large.

On the other hand, when a meta-analysis was performed comparing the use of HVE with LVE (Fig. 4), there was no significant difference (*P* = 0.40/CI -0.71[-2.47, 0.95]) in reducing the amount of aerosol produced in the environment, quantifying the magnitude of the effect, according to the Cohen scale [26], in medium.

### Certainty of evidence

The certainty of the evidence is represented in Tables 3 and 4.

In the subgroup analysis for distances less than or equal to 60 cm and greater than or equal to 120 cm from the oral cavity, contamination of the dental environment with the use of ultrasonic scaler was greater than without the use of ultrasonic scaler and the certainty of the evidence was considered moderate for both distances (Table 4).

**Table 4:** Contamination of the dental environment by aerosol produced during scaling with ultrasonic scaler

Certainty assessment							Number of patients		Efect		Certainty	Importance
Number of studies	Study design	Risk of bias	Inconsistency	Indirect evidence	Imprecision	Other considerations	Before ultrasonic scaling	During ultrasonic scaling	Relative (95% IC)	Absolute (95% IC)		
**Distance less than or equal to 60 cm from the oral cavity**												
3	Randomized clinical trials	not serious	not serious	not serious	serious^a^	none	49	49	-	mean **1.06 lower** (1.52 higher to 0.66 lower)	⨁⨁⨁◯ Moderate	IMPORTANT
**Distance greater than or equal to 120 cm from the oral cavity**												
3	Randomized clinical trials	not serious	not serious	not serious	serious^a^	none	37	37	-	mean **0.87 lower** (1.35 higher to 0.39 lower)	⨁⨁⨁◯ Moderate	IMPORTANT

Subtitle: CI- Confidence Interval

Explanation

a. Small sample for continuous data and wide confidence interval

When comparing the use of high-volume suction with the use of low-volume suction in reducing levels of contamination in the dental environment, the certainty of the evidence was considered low (Table 5), with no significant differences between these devices. Serious problems with inconsistency and imprecision were detected in the studies included in the meta-analyses.

**Table 5:** High Volume Suction (HVE) versus Low Volume Suction (LVE) in reducing the level of aerosol contamination

Certainty assessment							Number of patients		Efect		Certainty	Importance
Number of studies	Study design	Risk of bias	Inconsistency	Indirect evidence	Imprecision	Other considerations	High volume evacuation (HVE)	Low volume evacuation (LVE)	Relative (95% IC)	Absolute (95% IC)		
Distance less than or equal to 50 cm from the oral cavity												
3	Randomized clinical trials	not serious	serious^a^	not serious	serious^b^	none	98	98	-	mean **0.71 higher** (0.95 lower to 2.37 higher)	⨁⨁◯◯ Low	IMPORTANT

Subtitle: CI- Confidence Interval

Explanation

a. Considerable heterogeneity between studies (*p* < 0.05) and no overlapping confidence intervals

b. Small sample for continuous data and wide confidence interval

## Discussion

Aerosols and splashes are the main sources of environmental contamination during dental procedures [27]. This fact has become one of the biggest concerns among dentists during the COVID-19 pandemic. In order to review the evidence related to air contamination generated by the reach of the aerosol produced during the use of ultrasonic scaler for scaling and prophylaxis, a detailed search was carried out in six databases and five randomized controlled trials who met the inclusion criteria were found.

The high bacterial counts (log10 5.0 CFUs/mL) indicate that there is a worrying contamination of the air after the use of ultrasonic scaler, even when using a high volume suction combined with another device [22]. This contamination was shown in the first meta-analysis (Fig. 3) carried out on the results of randomized controlled studies [23–25], in which even with the use of high-volume suction, there was a significant difference in the increase of bacteria in the air (*P* < 0.00001/CI 0.99, [0.67, 1.31]).

Of the five randomized controlled trials included, only two [23, 24] found a statistically significant reduction in the mean CFUs (*p* < 0.001) collected during the use of ultrasonic scaler when using two different suction methods, where one used the high-pressure suction cannula volume attached directly to the ultrasonic pen [23], and the other the high volume suction combination added plus a high volume suction hose [24]. The other RCTs [21, 22] did not find significant differences (*p* > 0.05) between the two suction methods studied. However, the number of bacteria in the air tends to be higher when conventional suction devices are used, that is, low volume ones [25]. Furthermore, the high-volume suction device used separately, without any modification, does not appear to be as effective in reducing the amount of aerosol formed [21]. Despite this, when a meta-analysis (Fig. 4) was performed comparing the use of high and low volume suction devices in three RCTs [21, 23, 25], there was no significant difference in the amount of aerosol formed during the use of ultrasonic scaler.

As limitations of the study, a difference was observed in the methodologies used by the studies, which makes a more accurate comparison difficult, as the distance at which the agar plates are placed to collect the samples, the plate exposure time and the different dental environments, can influence the comparison of results. Two RCTs [22, 23] placed the sample collection plates six inches (15.24 cm) from the patient’s mouth and the others used different distances such as 40 and 150 cm [25]; 12 and 20 inches (approximately 30 and 50 cm) [21] and at three different distances between 2 and 4 feet (approximately 60–120 cm) (24). Regarding the exposure time of the plaque during the use of ultrasound, there was a variation between 5 min [23, 25] and 20 min [24]. Therefore, the shorter the plate exposure time and the greater the distance, the lower the chance of CFU collection. And regarding the dental environments just one study used a multi-chair environment [24].

Another limitation of the study refers to the fact that it was not possible to assess publication bias as only five studies were included for meta-analysis, with low power to detect possible bias.

## Conclusions

There is an increase in the concentration of bioaerosol in the dental environment during the use of ultrasonic scaler in scaling/prophylaxis, reaching up to 2 m away from the patient’s mouth.

The use of good suction, whether low volume, high volume or a combination of different devices, can be effective in reducing air contamination in the dental environment, with no important difference between different types of suction devices.

Final considerations: To minimize the risk of infection for the operator, it is recommended to use adequate precautions, such as the use of adapted masks. And to minimize the risk of cross-infection, especially between patients, and contamination of surfaces, it is recommended to space appointments by at least 30 min and always use suction devices respectively.

## Data Availability

The datasets generated and/or analyzed during the current study are available from the corresponding author on reasonable request.

## Abbreviations

CFU: Colony forming units
CI: Confidence intervals
COVID-19: Coronavirus disease 2019
CDC: Centers for Disease Control and Prevention
HVE: High Volume Suction
LVE: Low Volume Suction
PEO: Population, Exposition, and Outcome
RCTs: Randomized controlled trials
RoB 2: Risk of Bias 2
SARS-CoV-2: Severe acute respiratory syndrome coronavirus 2

## References

[1] Gralinski LE, Menachery VD (2020). Return of the coronavirus: 2019-nCoV. Viruses.

[2] Centers for Disease Control and Prevention (CDC). COVID-19 Overview and Infection Prevention and Control Priorities in non-U.S. 2021.

[3] Komperda J, Peyvan A, Li D, Kashir B, Yarin AL, Megaridis CM (2021). Computer simulation of the SARS-CoV-2 contamination risk in a large dental clinic. Phys Fluids.

[4] Adhikari U, Chabrelie A, Weir M, Boehnke K, McKenzie E, Ikner L (2019). A Case Study evaluating the risk of infection from Middle Eastern Respiratory Syndrome Coronavirus (MERS-CoV) in a hospital setting through Bioaerosols. Risk Anal.

[5] Yu ITS, Li Y, Wong TW, Tam W, Chan AT, Lee JHW (2004). Evidence of Airborne Transmission of the severe Acute Respiratory Syndrome Virus. N Engl J Med.

[6] Micik RE, Miller RL, Mazzarella MA, Ryge G (1969). Studies on Dental Aerobiology: I. Bacterial Aerosols Generated during Dental procedures. J Dent Res.

[7] Larato DC, Ruskin PF, Martin A (1967). Effect of an Ultrasonic Scaler on Bacterial counts in Air. J Periodontol.

[8] Holbrook WP, Muir KF, MacPhee IT, Ross PW (1978). Bacteriological investigation of the aerosol from ultrasonic scalers. Br Dent J.

[9] Graziani F, Izzetti R, Lardani L, Totaro M, Baggiani A (2021). Experimental evaluation of Aerosol Production after Dental Ultrasonic Instrumentation: an analysis on fine particulate matter perturbation. IJERPH.

[10] Balcoș CB, Săveanu I, Bobu L, Bosînceanu D, Bolat M, Grădinaru I (2019). The risk of contamination through ultrasonic scaling. Romanian J Oral Rehabilitation.

[11] International prospective register of systematic reviews. 2020.

[12] Higgins J, Thomas J, Chandler J, Cumpston M, Li T, Page M et al. Cochrane Handbook for Systematic Reviews of Interventions version 6.3. 2022.

[13] Page MJ, McKenzie JE, Bossuyt PM, Boutron I, Hoffmann TC, Mulrow CD et al. The PRISMA 2020 statement: an updated guideline for reporting systematic reviews. BMJ. 2021;:n71. 10.1136/bmj.n71 PMC8005924 33782057

[14] OpenGrey open access database. 2021.

[15] Medical S. Headings. 2021.

[16] Health S. Descriptors. 2021.

[17] Endnote. web software. 2021.

[18] Higgins JPT, Altman DG, Gotzsche PC, Juni P, Moher D, Oxman AD (2011). The Cochrane collaboration’s tool for assessing risk of bias in randomised trials. BMJ.

[19] Review Manager (RevMan). [Computer program]. Version 5.4.1, The Cochrane Collaboration. 2020.

[20] Guyatt GH, Oxman AD, Sultan S, Glasziou P, Akl EA, Alonso-Coello P (2011). GRADE guidelines: 9. Rating up the quality of evidence. J Clin Epidemiol.

[21] Desarda H, Gurav A, Dharmadhikari C, Shete A, Gaikwad S. Efficacy of high-volume Evacuator in Aerosol reduction: truth or myth? A clinical and microbiological study. J Dent Res. 2014;Dental Clinics:Dental Prospects; eISSN 20082118. 10.5681/joddd.2014.032 PMC4206761 25346838

[22] Holloman JL, Mauriello SM, Pimenta L, Arnold RR (2015). Comparison of suction device with saliva ejector for aerosol and spatter reduction during ultrasonic scaling. J Am Dent Association.

[23] King TB, Muzzin KB, Berry CW, Anders LM (1997). The effectiveness of an Aerosol reduction device for Ultrasonic Sealers. J Periodontol.

[24] Suprono MS, Won J, Savignano R, Zhong Z, Ahmed A, Roque-Torres G (2021). A clinical investigation of dental evacuation systems in reducing aerosols. J Am Dent Association.

[25] Timmerman MF, Menso L, Steinfort J, Van Winkelhoff AJ, Van Der Weijden GA (2004). Atmospheric contamination during ultrasonic scaling. J Clin Periodontol.

[26] Cohen J Statistical power analysis for the behavioral sciences. Academic press., Harrel SK, Barnes JB, Rivera-Hidalgo F. Aerosol and splatter contamination from the operative site during ultrasonic scaling. The Journal of the American Dental Association. 1998;129:1241–9. 10.14219/jada.archive.1998.0421 9766105

